# Agreement Between Invasive Wire-Based and Angiography-Based Vessel Fractional Flow Reserve Assessment on Intermediate Coronary Stenoses

**DOI:** 10.3389/fcvm.2021.707454

**Published:** 2021-06-30

**Authors:** Chun-Chin Chang, Yin-Hao Lee, Ming-Ju Chuang, Chien-Hung Hsueh, Ya-Wen Lu, Yi-Lin Tsai, Ruey-Hsing Chou, Cheng-Hsueh Wu, Tse-Min Lu, Po-Hsun Huang, Shing-Jong Lin, Robert-Jan van Geuns

**Affiliations:** ^1^Division of Cardiology, Department of Medicine, Taipei Veterans General Hospital, Taipei, Taiwan; ^2^Institute of Clinical Medicine and Cardiovascular Research Center, National Yang Ming Chiao Tung University, Taipei, Taiwan; ^3^Department of Cardiology, Thoraxcenter, Erasmus Medical Center, Rotterdam, Netherlands; ^4^Department of Critical Care Medicine, Taipei Veterans General Hospital, Taipei, Taiwan; ^5^Taipei Heart Institute, Taipei Medical University, Taipei, Taiwan; ^6^Cardiology Department, Radboud University Medical Center, Nijmegen, Netherlands

**Keywords:** fractional flow reserve, coronary artery disease, percutaneous coronary intervention, resting full-cycle ratio, vessel fractional flow reserve

## Abstract

**Background:** Angiography-based functional assessment of coronary stenoses emerges as a novel approach to assess coronary physiology. We sought to investigate the agreement between invasive coronary wire-based fractional flow reserve (FFR), resting full-cycle ratio (RFR), and angiography-based vessel FFR (vFFR) for the functional assessment of coronary stenoses in patients with coronary artery disease.

**Materials and Methods:** Between Jan 01, 2018, and Dec 31, 2020, 298 patients with 385 intermediate lesions received invasive coronary wire-based functional assessment (FFR, RFR or both) at a single tertiary medical center. Coronary lesions involving ostium or left main artery were excluded. vFFR analysis was performed retrospectively based on aortic root pressure and two angiographic projections.

**Results:** In total, 236 patients with 291 lesions were eligible for vFFR analysis. FFR and RFR were performed in 258 and 162 lesions, respectively. The mean FFR, RFR and vFFR value were 0.84 ± 0.08, 0.90 ± 0.09, and 0.83 ± 0.10. vFFR was significantly correlated with FFR (*r* = 0.708, *P* < 0.001) and RFR (*r* = 0.673, *P* < 0.001). The diagnostic performance of vFFR vs. FFR was accuracy 81.8%, sensitivity 77.4%, specificity 83.9%, positive predictive value 69.9%, and negative predictive value 88.5%. The discriminative power of vFFR for FFR ≤ 0.80 or RFR ≤ 0.89 was excellent. Area under the receiver operating characteristic curve (AUC) was 0.87 (95% CI:0.83–0.92) for FFR and 0.80 (95% CI:0.73–0.88) for RFR.

**Conclusion:** Angiography-based vFFR has a substantial agreement with invasive wire-based FFR and RFR in patients with intermediate coronary stenoses. vFFR can be utilized to assess coronary physiology without a pressure wire in a *post hoc* manner.

## Introduction

Fractional Flow Reserve (FFR) is an invasive wire-based physiology index for assessing the functional significance of coronary stenoses during adenosine-induced hyperemia. The FAME (Fractional Flow Reserve vs. Angiography for Multivessel Evaluation) and FAME 2 trials have demonstrated that FFR-guided percutaneous coronary intervention (PCI) strategy is associated with a significantly lower rate of cardiovascular events in patients with stable coronary artery disease (1, 2).

Performing PCI procedures in accordance to FFR recommendations was associated with better clinical outcomes (3).

FFR or an instantaneous wave-free ratio (iFR) is the current standard of care for assessing the hemodynamic relevance of intermediate-grade coronary stenoses and recommended by the European guidelines (4, 5). Nevertheless, the adoption rate of FFR remains low in the real-world practice for several reasons (e.g., prolonged procedure time, side effects of adenosine, patient-related discomfort, or cost-effective issues) (6).

Novel physiological indices have been developed to reduce the procedural and invasive aspects for functional assessment of coronary artery disease. The resting full-cycle ratio (RFR), a novel non-hyperemic index, is developed to identify the lowest distal coronary pressure to an aortic pressure ratio (Pd/Pa) within the entire cardiac cycle and is diagnostically equivalent to iFR (7). A recent study reported all resting pressure-derived physiological indices may be used as invasive tools to guide treatment strategy in patients with coronary artery disease (8).

Vessel FFR (vFFR) is a kind of angiography-based FFR assessment calculated by a dedicated software using two contrast filled angiograms to generate a three-dimensional (3D) model of coronary artery and assesses pressure drop (9). The clinical value of vFFR in diagnosis and management of coronary artery disease is currently being investigated. This study aimed to examine the agreement between invasive coronary wire-based FFR, RFR, and vFFR for the functional assessment of coronary stenoses.

## Methods

### Study Design and Participants

The present study is an observational, retrospective, single-center study comparing vFFR with wire-based FFR and RFR. Between Jan 01, 2018 and Dec 31, 2020, patients presenting with chronic coronary syndrome, unstable angina or non-ST elevation myocardial infarction who underwent pre-procedural FFR or RFR assessment were eligible. Left main artery disease or ostial lesions were excluded. The study was approved by the research ethics committee of the Taipei Veterans General Hospital and was conducted in accordance with the Declaration of Helsinki.

### Invasive Physiological Assessment

Procedures were performed according to standard local clinical practice in the catheterization laboratory. FFR/RFR was measured using 6 or 7 French guiding catheters and a 0.014 PressureWire™ X Guidewire (Abbott Vascular Inc., Santa Clara, CA,) positioned distal to the target lesion. The location of pressure sensor was recorded by cineangiography. QUANTIEN™ or OPTIS™ Integrated System (Abbott Vascular Inc., Santa Clara, CA,) was used for FFR/RFR measurement. RFR was defined as the lowest Pd/Pa ratio during the entire cardiac cycle (7). FFR was measured under hyperemia by intravenous adenosine infusion at 140 μg/kg/min or intracoronary (IC) bolus injection of adenosine. The dose of IC adenosine initiated from 60 or 120 μg for right coronary artery (RCA) and 120 μg for left coronary artery. IC adenosine was up titrated gradually to 180, 240, 360 or 480 μg. At least two FFR measurements were performed to confirm the FFR value. The lowest FFR value was used after multiple measurements.

### vFFR Computation

**Figure 1:**
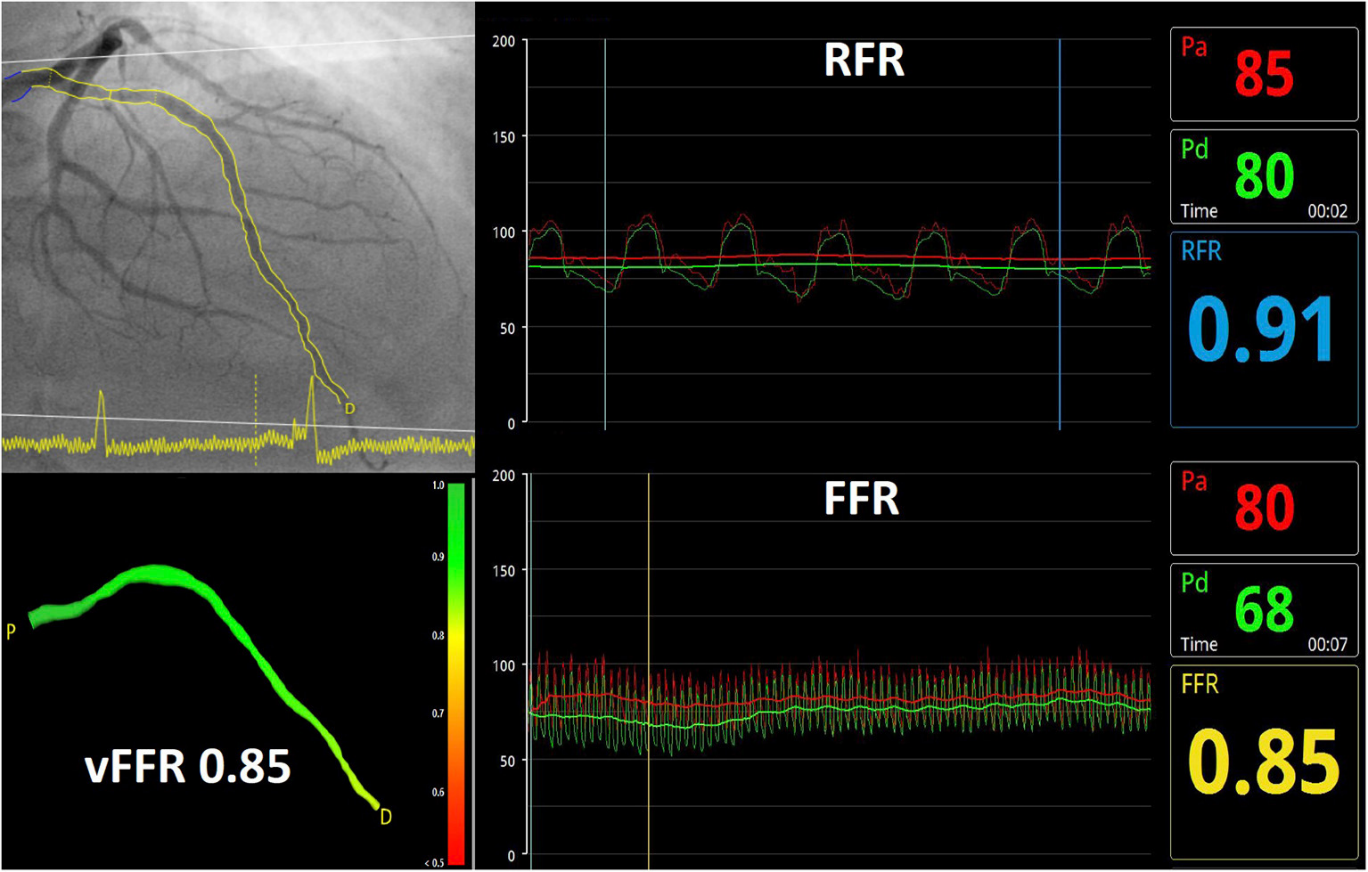
Illustration of a vFFR analysis.

vFFR computation was performed using CAAS Workstation (Version 8.2; Pie Medical Imaging, Maastricht, the Netherlands) by three independent analysts (CC Chang, YH Lee and MJ Chuang) blinded to FFR and RFR values. All analysts were trained by Pie Medical Imaging. To perform the vFFR analysis, image acquisition and processing were reported previously (9). Briefly, two angiograms with at least 30 degrees difference in rotation/ angulation are required to create a 3D reconstruction of the coronary artery. The software contour detection was performed semiautomatically, delineating the vessel contour from the ostium to a distal position with manual correction when needed. The location of invasive pressure wire for FFR or RFR measurement was identified in the angiogram and was utilized to match the distal contour of vFFR measurement. 3D quantitative coronary angiogram (QCA) and vFFR value were automatically generated. A representative case of vFFR assessment was shown in Figure 1.

### Statistical Methods

Categorical variables were presented as percentages and numbers. Continuous variables were presented as mean ± standard deviation. Pearson's correlation coefficient was used to evaluate the relationship between vFFR, FFR, and RFR. Agreement between vFFR and FFR was assessed by Bland-Altman plots and intraclass correlation coefficients (ICC). The receiver operating characteristic (ROC) graph and the area under the curve (AUC) was used to estimate the discriminative value of vFFR for functionally significant coronary lesions (FFR ≤ 0.80 or RFR ≤ 0.89). A two-sided *p*-value of < 0.05 was considered to indicate statistical significance. Data were analyzed using SPSS software (version 25, SPSS, Chicago, Illinois, USA).

## Results

**Figure 2:**
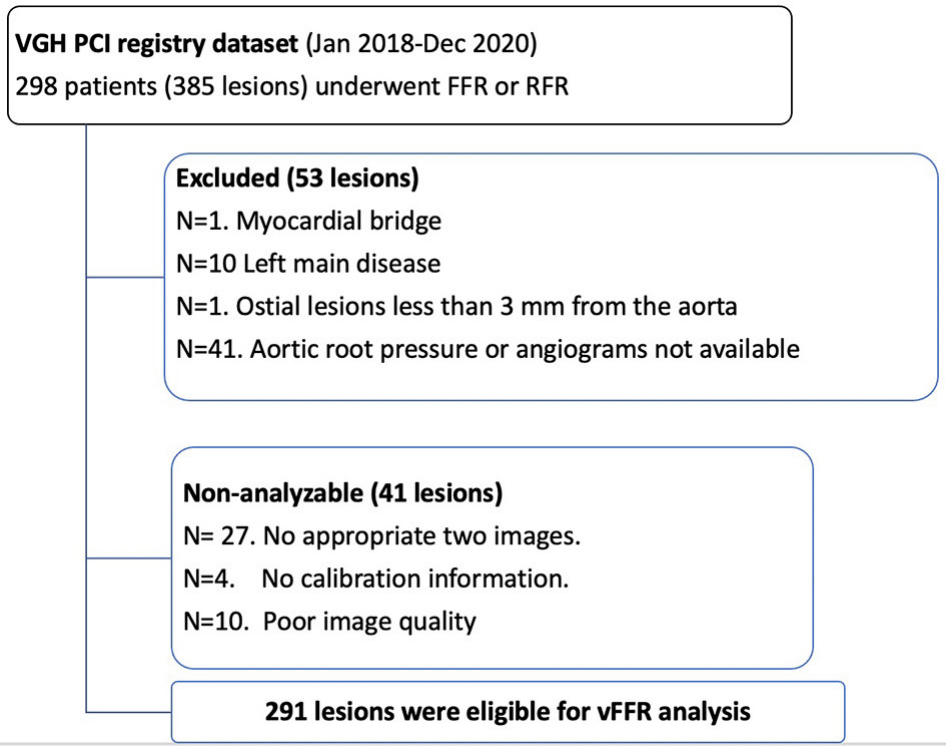
Study flow.

A total of 385 lesions in 298 patients were interrogated. Fifty-three lesions were excluded due to involving left main coronary artery, ostial lesion, myocardial bridge or aortic root pressure/angiogram was not available. vFFR was non-analyzable in 41 lesions, mainly due to no appropriate two angiographic projections or poor image quality. After all, 291 lesions from 236 patients were eligible for vFFR analysis (Figure 2). FFR and RFR were performed in 258 and 162 lesions, respectively. The baseline characteristics of eligible patients are shown in Table 1. The mean age was 67.9 ± 11.3 years, 73.3% were male, and 56.4% presented with the chronic coronary syndrome. The majority of physiology assessment was performed in the left anterior descending artery (LAD) (64.0%). 3D QCA data is provided in Table 2. Mean minimal lumen diameter, percent diameter stenosis, and obstruction length were 1.60 ± 0.43 mm, 42.9 ± 10.9% and 24.2 ± 16.1 mm.

**Table 1:** Baseline characteristics of patients.

**Baseline characteristics**	**All patients (*n* = 236)**
Age (years)	67.9 ± 11.3
**Sex**	
Male	173 (73.3%)
Female	63 (26.7%)
**Medical history**	
Hypertension	182 (77.1%)
Diabetes mellitus	111 (47.0%)
Peripheral vascular disease	9 (3.8%)
Chronic kidney disease	65 (27.5%)
Previous myocardial infarction	9 (3.8%)
Previous percutaneous coronary intervention	77 (32.6%)
Previous coronary artery bypass grafting	4 (1.7%)
**Clinical presentation**	
Chronic coronary syndrome	133 (56.4%)
Unstable angina	95 (40.3%)
Non-ST elevation myocardial infarction	8 (3.4%)
**Number of diseased vessels**	
Single-vessel disease	84 (35.6%)
Two-vessel disease	92 (39.0%)
Three-vessel disease	60 (25.4%)

**Table 2:** Baseline characteristics of lesions studied.

**Baseline characteristics**	**All lesions (*n* = 291)**
**Vessel studied**	
Left anterior descending artery	186 (64.0%)
Left circumflex	45 (15.4%)
Right coronary artery	60 (20.6%)
**3D Quantitative coronary angiography**	
Reference vessel diameter (mm)	2.84 ± 0.69
Minimal lumen diameter (mm)	1.60 ± 0.43
Percent diameter stenosis (%)	42.9 ± 10.9
Percent area stenosis (%)	66.3 ± 12.3
Obstruction length (mm)	24.2 ± 16.1

**Figure 3:**
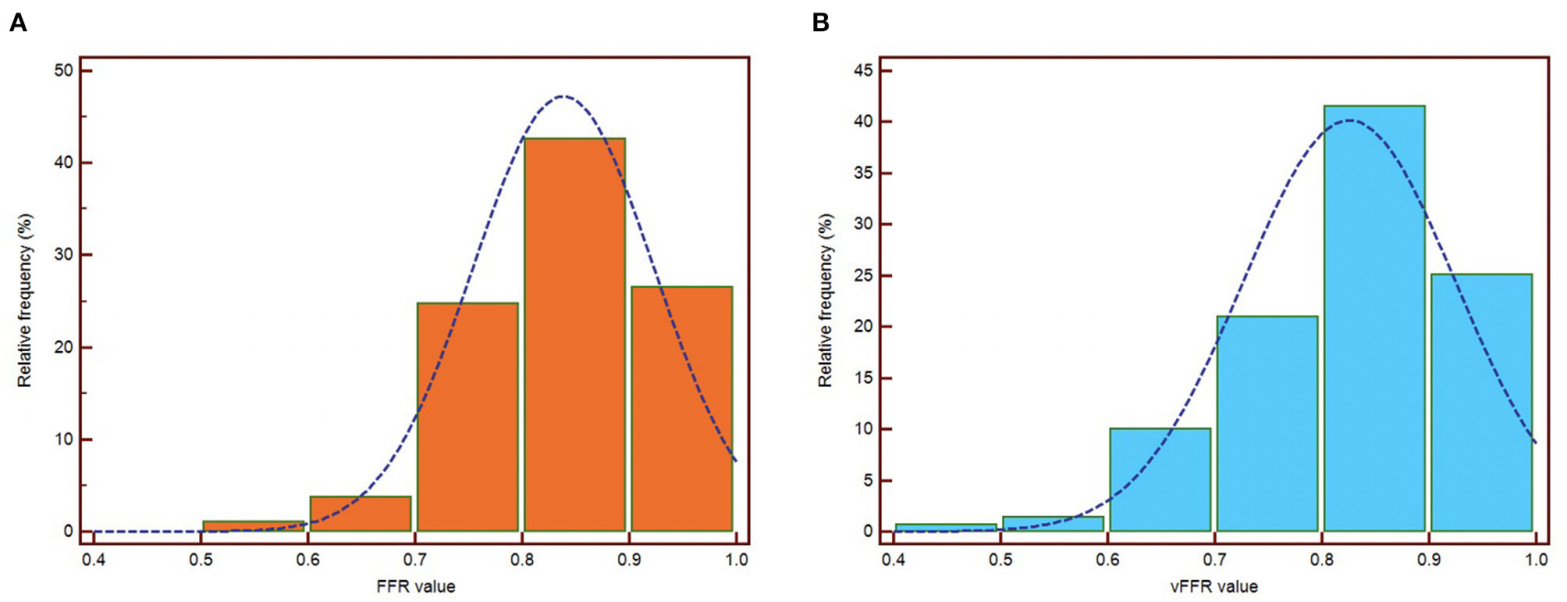
Distribution of values for FFR **(A)** and vFFR **(B)**.

**Figure 4:**
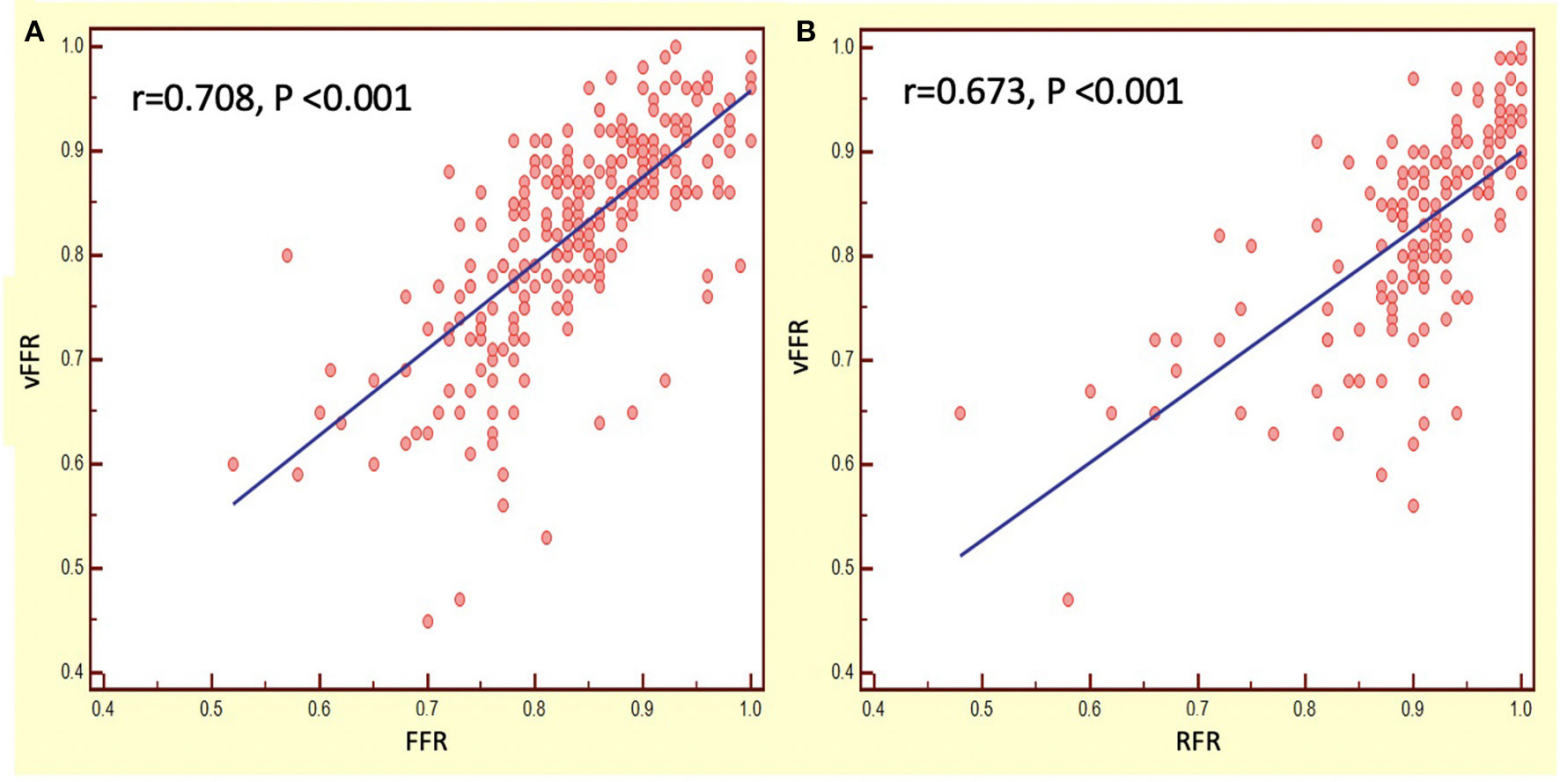
Correlation between vFFR, RFR, and FFR. **(A)** vFFR vs. FFR; **(B)** vFFR vs. RFR.

**Figure 5:**
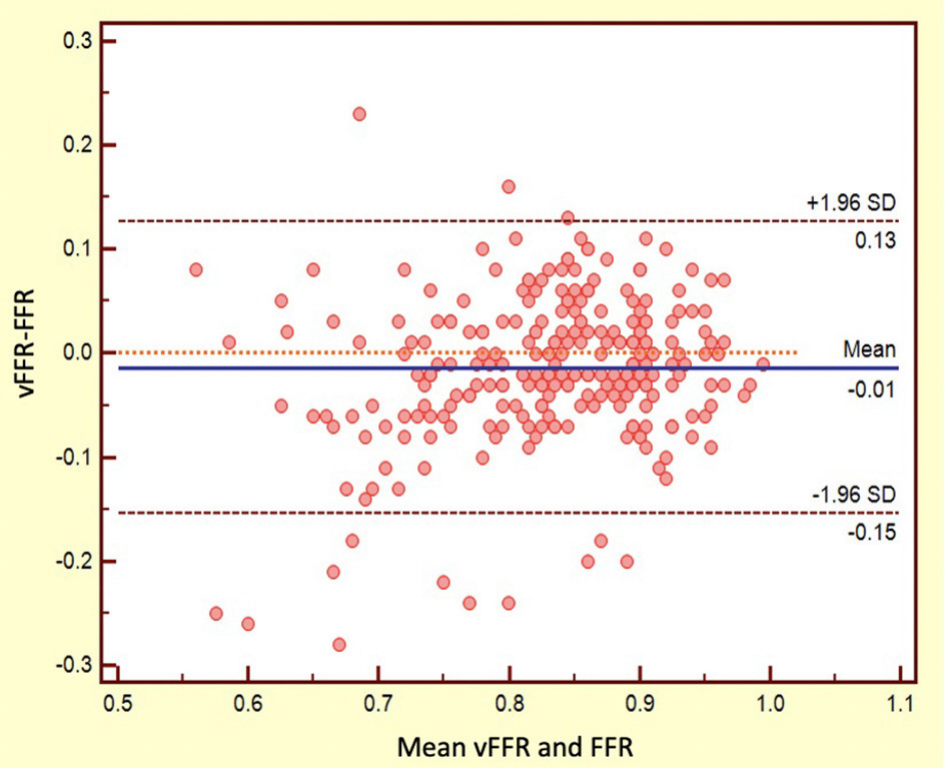
Bland–Altman plots: vFFR vs. FFR.

**Figure 6:**
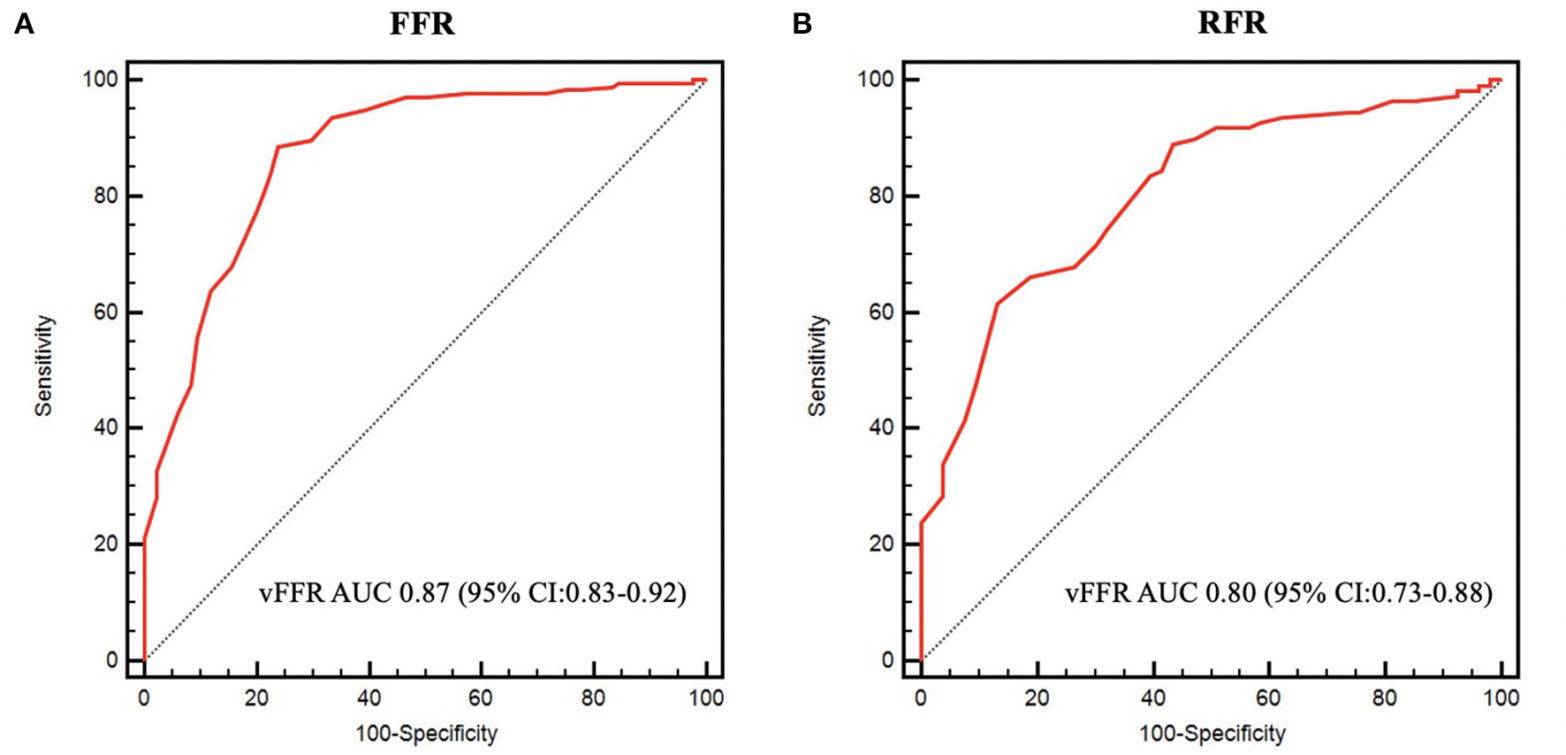
Receiver operating characteristic curves. **(A)** ROC curve of vFFR for FFR ≤ 0.80. **(B)** ROC curve of vFFR for RFR ≤ 0.89.

The mean FFR, RFR, and vFFR value were 0.84 ± 0.08, 0.90 ± 0.09, and 0.83 ± 0.10 respectively. The distribution of values of FFR and vFFR are shown in Figure 3. vFFR had a high positive correlation with FFR (*r* = 0.708, *P* < 0.001) and a moderate positive correlation with RFR (*r* = 0.673, *P* < 0.001) (Figure 4). The Bland-Altman plots displaying vFFR had a good agreement with FFR [mean difference = −0.01 (95% CI: −0.15, 0.13), ICC = 0.70 (95% CI: 0.63–0.76), *P* < 0.001] (Figure 5). Using the same cut-off value of ≤ 0.80 to define a functionally significant lesion for vFFR, the diagnostic concordance between vFFR and FFR was 81.8 and 74.1% between vFFR and RFR (Table 3). The diagnostic performance of vFFR vs. FFR was diagnostic accuracy 81.8%, sensitivity 77.4%, specificity 83.9%, positive predictive value 69.9%, and negative predictive value 88.5%. The discriminative power of vFFR for functionally significant coronary lesions (FFR ≤ 0.80 or RFR ≤ 0.89) was excellent. AUC was 0.87 (95% CI:0.83–0.92) for FFR and 0.80 (95% CI:0.73–0.88) for RFR (Figure 6).

**Table 3:** Diagnostic concordance between vFFR, FFR, and RFR.

	**FFR > 0.8 (*n* = 174)**	**FFR ≤ 0.8 (*n* = 84)**
**vFFR** **>** **0.8** (*n* = 165)	True negative 56.6% (146/258)	False negative 7.4% (19/258)
**vFFR** **≤** **0.8** (*n* = 93)	False positive 10.9% (28/258)	True positive 25.2% (65/258)
	**RFR** **>** **0.89 (*****n*** **=** **109)**	**RFR** **≤** **0.89 (*****n*** **=** **53)**
**vFFR** **>** **0.8** (*n* = 105)	True negative 53.1% (86/162)	False negative 11.7% (19/162)
**vFFR** **≤** **0.8** (*n* = 57)	False positive 14.2% (23/162)	True positive 21.0% (34/162)

*FFR, fractional flow reserve; RFR, resting full-cycle ratio; vFFR, vessel fractional flow reserve*.

### Impact of Gray Zone on Diagnostic Performance

A total of 149 lesions had a vFFR value in the gray zone (95% sensitivity; 97% specificity) (0.75 ≤ vFFR ≤ 0.89), whereas 109 lesions had a vFFR value outside the gray zone (vFFR<0.75 or vFFR>0.89). The diagnostic performance of vFFR was outstanding for FFR ≤ 0.80 when vFFR was outside the gray zone [AUC: 0.94 (95% CI:0.869–0.99), *P* < 0.001]. However, the diagnostic performance of vFFR was only acceptable when vFFR was in the gray zone [AUC: 0.70 (95% CI:0.60–0.80), *P* < 0.001], assuming that FFR is always accurate.

## Discussion

The present study investigated the agreement between invasive coronary wire-based FFR, RFR, and angiography-based vFFR for the functional assessment of coronary stenoses. Our study showed that vFFR had a good correlation and agreement with FFR and RFR. vFFR had an excellent discriminative power to detect functionally significant coronary lesions without the use of a pressure wire.

The correlation between vFFR and FFR was firstly reported in the FAST study (9). The FAST-study is an observational, retrospective, single-center study which showed an excellent linear correlation between FFR and vFFR (*r* = 0.89, *p* < 0.001) with good reproducibility (inter-observer variability, *r* = 0.95, *p* < 0.001). The diagnostic accuracy of vFFR in identifying lesions with an FFR ≤ 0.80 was good, with AUC 93% (95% CI: 88–97%). The demographics and lesion severity of our study was similar to the FAST study. Our study results confirmed the observation in the FAST study that vFFR had a high positive correlation with FFR. Nevertheless, in the FAST study, the correlation coefficient and AUC of vFFR vs. FFR were slightly higher than the present study which may be attributed to a more stricter image acquisition requirement. It is worth to note that Pizzato et al. reported the feasibility of vFFR analysis was only 35.1% with a weak correlation with FFR by using an imaging database maintained in a core lab (10). However, in the study mentioned above, both pre- and post-PCI angiograms were analyzed and collected from multiple sites without a pre-specified image acquisition. In contrast, the feasibility of vFFR analysis was higher in our study. The potential explanation is that coronary angiograms are routinely acquired using a biplane x-ray system with regular projections in our catheterization laboratory which may increase the analyzability of vFFR. Of note, there was a higher prevalence of LAD lesions than left circumflex (LCX) and RCA lesions in our study. Among 41 non-analyzable lesions, the distribution was 19, 16, and 6 for LAD, LCX and RCA, respectively. The analyzability was lower in LCX (73.8%) than LAD (90.7%) and RCA (90.9%) lesions in our cohort. This observation may be attributed to the vessel tortuosity of LCX which limited the vFFR analysis. Therefore, further studies are warranted to evaluate the software following a pre-specified imaging acquisition protocol and compare the result of FFR at the same time to validate the diagnostic performance of vFFR.

In the VALIDATE RFR study, RFR was significantly correlated with FFR (*r* = 0.746, *p* < 0.001), and the diagnostic performance of RFR vs. FFR was nearly identical with iFR vs. FFR (diagnostic accuracy 81.3%, sensitivity 71.5%, specificity 88.0%, positive predictive value 80.6%, negative predictive value 81.6%, AUC of 88.1%) (7). In addition, the prognostic value of RFR has been reported recently. RFR was significantly associated with the risk of 2-year vessel-oriented composite outcome (a composite of cardiac death, vessel-related myocardial infarction, and vessel-related ischemia-driven revascularization) (8). In view of the increasing use of wire-based or wireless non-hyperemic indices recently in clinical practice to avoid the side effect of adenosine infusion during FFR assessment, we further explored the correlation between vFFR and RFR in our study which has not yet been reported in the literature. Our study showed that vFFR had a moderate positive correlation and a moderate diagnostic agreement with RFR. However, further studies may be needed to confirm our findings from a large study population.

A recently published meta-analysis has shown that the diagnostic performance of angiography derived FFR is good with high sensitivity and specificity with measured FFR as reference (11). In addition, there are no differences in accuracy for detecting functionally significant lesions between different software packages including quantitative flow ratio (QFR), vFFR and FFR_angio_. The diagnostic performance of QFR has been broadly investigated and reported (12, 13). QFR also had a good diagnostic correlation with FFR in assessing non-culprit lesions in patients with acute coronary syndrome and multivessel disease (14, 15). Although QFR and vFFR are both angiography-derived FFR, they are computed using different fluid dynamics models (16). Data on the diagnostic performance of vFFR remains to be further explored. As such, the scope of the present study was mainly focused on the agreement of vFFR and the pressure wire-based FFR/RFR in relatively low risk patients with intermediate coronary stenoses.

The physiological assessment of coronary stenoses remains underused despite accumulating evidence supporting its clinical benefit. The feasibility and excellent diagnostic performance of angiography-based FFR may increase the adoption rate of coronary physiological assessment in real-world practice. In this context, angiography-based FFR may play a role in specific clinical settings, such as non-culprit lesion assessment in ST elevation myocardial infarction (14, 17), post coronary stenting (18, 19), risk stratification (20) or event adjudication in clinical trials (21, 22).

To date, FFR remain the gold standard for assessing coronary physiology. Similar to FFR derived from coronary computed tomography angiography (FFR_CT_), angiography-based FFR may be utilized as a surrogate for invasive FFR. In our study, the mean difference between vFFR and FFR was only 0.01, but the 95% CI was relatively large which was still consistent with FFR_CT_ data (23) and abovementioned meta-analysis (11). As such, a hybrid approach (e.g., angiography-based FFR first and confirmed by invasive FFR if required) is recommended, especially when angiography-based FFR is in the gray-zone.

### Strengths and Limitations

Several limitations have to be acknowledged in our study. First of all, this is a retrospective analysis, and therefore, coronary angiograms were obtained based on routine clinical practice. A dedicated imaging acquisition protocol was not implemented in our study. Second, the use of physiological assessment was at the discretion of treating physicians and potential selection bias could not be avoided. Third, the results from a single center experience with a limited sample size need to be confirmed in further studies. Fourth, coronary stenoses involving the left main or ostial lesions were excluded, and the utility of vFFR in these specific anatomical locations warrants further investigations. Lastly, an independent core lab was lacking in our study for vFFR computation or FFR and RFR waveform interrogation.

In conclusion, angiography-based vFFR has a substantial agreement with invasive wire-based FFR and RFR in patients with intermediate coronary artery stenoses. vFFR can be utilized to assess coronary physiology without a pressure wire in a *post hoc* manner. The clinical implication and association with outcomes warrant further investigation.

## Data Availability Statement

The raw data supporting the conclusions of this article will be made available by the authors, without undue reservation.

## Ethics Statement

The studies involving human participants were reviewed and approved by the research ethics committee of the Taipei Veterans General Hospital. Written informed consent for participation was not required for this study in accordance with the national legislation and the institutional requirements.

## Author Contributions

C-CC, P-HH, S-JL, and R-JG contributed to the conception and design of the study. C-CC, Y-HL, M-JC, C-HH, Y-WL, Y-LT, R-HC, C-HW, and T-ML contributed to data collection. C-CC, Y-HL, and M-JC analyzed and interpreted the data. C-CC, Y-HL, and M-JC drafted the report, which was critically revised for important intellectual content by T-ML, P-HH, S-JL, and R-JG. All authors have participated in the work and have reviewed and agree with the content of the article, approved the final version of the report.

## Conflict of Interest

The authors declare that the research was conducted in the absence of any commercial or financial relationships that could be construed as a potential conflict of interest.
